# Investigations of the electrochemical performance and filling effects of additives on electroplating process of TSV

**DOI:** 10.1038/s41598-020-66191-7

**Published:** 2020-06-08

**Authors:** Houya Wu, Yan Wang, Zhiyi Li, Wenhui Zhu

**Affiliations:** 1 State Key Laboratory of High Performance Complex Manufacturing, Central South University, Changsha, 410083 China; 2 College of Mechanical and Electrical Engineering, Central South University, Changsha, 410083 China

**Keywords:** Mechanical engineering, Electrochemistry

## Abstract

Through silicon via (TSV) is one of the most important technologies used in three dimension (3D) packaging. The void-free filling of TSV can be achieved by adding additives into the electrolyte bath during the electrodeposition process. This paper focuses on the effects of three types of commercial additives (the suppressor, the leveler and the accelerator) and analyses additives’ interaction on electroplating through experimental investigations. The results showed that the suppressor, the leveler and the accelerator all have chemical behaviour of inhibition in different degrees to the copper electroplating. The interaction experiments of additives in pairs indicated that the suppressor absorbed on the cathode surface was gradually displaced by the accelerator as the concentration of the accelerator increased; the accelerator and the leveler presented a competitive adsorption relationship; the suppressor and the leveler had a synergistic effect for electroplating inhibition especially under high potential and low suppressor concentration. Experiments of micro via filling by electrodeposition have been conducted to investigated the effects of singular additive and multiple additives on the filling process of the micro vias.

## Introduction

Nowadays, as the trend for the electronic devices manufacture becomes more integrated, the size of the electronic devices keeps diminishing. This makes three dimension (3D) packaging technology become increasingly prevalent^1–4^. Owing to the merits of high density, low energy consumption, high electrical performance and low RC delay, through silicon via (TSV) becomes the key technology in the 3D package^5^ in recent years. TSV, usually filled by copper (Cu), is typically achieved by electrodeposition^6,7^. As the micro structure of the TSV is too small (normally 5 to 40 micro-meters in width and 10 to 200 micro-meters in depth), non-uniform distribution of flow field, concentration field and electric field appears in the local region of the micro via, which leads to non-uniform filling rate between the opening and bottom of the TSV, especially of TSVs with high aspect ratio (AR)^8–13^. The non-uniformity may cause filling defects, voids and seams, and eventually result in poor electrical properties and reliability problems for TSVs^14–18^.

The principle of void-free filling of the micro via is to inhibit the filling rate at the via opening and accelerate the filling rate at the via bottom^19–21^. An effective way to accomplish this is adding additives and halogen ions into the plating bath, which changes the copper ions deposition rate on the cathode surface^5,9,22,23^. Additives are typically divided into three types: suppressor, accelerator and leveler^24,25^. The halogen ions (Cl^−^ in most cases)^26,27^ adsorb on the cathode surface and form cuprous electron bridges and bind themselves with the additives, leading to inhibition or acceleration of the copper electrodeposition^28–30^. Ko *et al*.^31^ achieved void-free deposition with polyethylene glycol (PEG) as suppressor. It was found that the inhibition effect of Cl^−^–Cu^+^–PEG complexes was governed not only by PEG concentration but also by PEG molecular weight. Moffat *et al*. studied the behaviour of bis (3-sulfopropyl) disulfide (SPS) as accelerator, and found that the effect of SPS on electrodeposition was potential dependent^32^. Hasegawa *et al*. investigated the effect of Janus green B (JGB) as leveler on micro via filling, and reached the conclusion that JGB inhibits the overfill phenomenon in the later stages of the deposition process, but the inhibition become greater with higher concentrations of JGB, resulting in void formation^33^. Akolkar and Landauz^34^ studied the interactions of PEG and SPS and proposed a competitive adsorption theory between PEG and SPS. It was clear that PEG adsorbed and polarized the electrode earlier than SPS, and was later on replaced completely by SPS on the electrode. Dow *et al*. found that PEG and JGB interacted and produced a synergistic inhibition effect on copper electrodeposition^35^. Although many studies^36–43^, have discussed the mechanism of the additives’ behaviours and their effects on TSV deposition, systematic investigations on additives’ interactions have rarely been found.

In this study, the suppressor (AESS), accelerator (M) and leveler (PNI) are purchased from Jiangsu Mengde New Materials Technology Co., Ltd, and their interaction effects on electrodeposition were investigated. Systematic experiments regarding to each additive separately and additives interaction were performed respectively. The electrochemistry behaviours of the additives under different current density were quantitatively characterized using chronoamperometry (CA) test. Experiments of micro via filling by electrodeposition were conducted, and the results were obtained by a scanning electron microscope (SEM).

## Experimental

**Figure 1:**
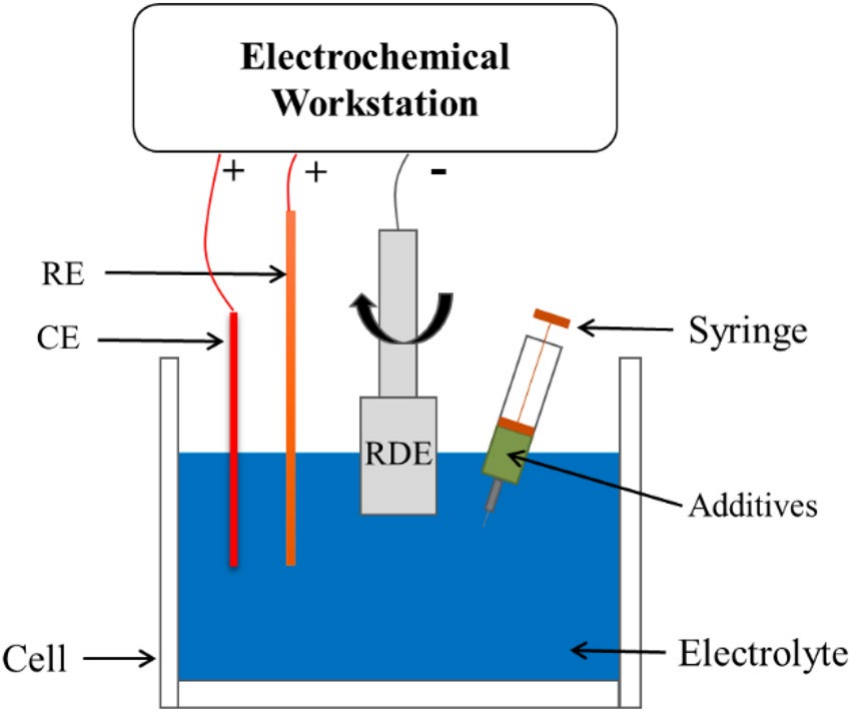
Schematic of the RDE setup for Chronoamperometry test (The additives were injected into base electrolyte using a syringe).

In order to investigate the effects of additives on the TSV filling process, the electrochemistry behaviours of the additives were quantitatively characterized using chronoamperometry (CA) test. Three additives, i.e. AESS, M and PNI were used as the suppressor, the accelerator and the leveler, respectively. The recorded current was used to analyse the effects of additives during the electrodeposition process for TSV filling. The CA measurements were conducted in a three-electrode cell with the base electrolyte (containing Cu^2+^ 0.63 mol/L, CH_3_SO_3_^−^ 0.21 mol/L, Cl^−^ 50 ppm) and varying concentration of the additives, as shown schematically in Fig. 1. The working electrode was a platinum rotating disc electrode (RDE) (99.9% purity, and 1.5 mm radius surrounded by a PTFE shroud). The counter electrode (CE) was a platinum wire (99.9% purity, 1 cm length, and 1 cm radius). A mercurous sulfate electrode (MSE) was used as reference electrode (RE). All electrodes were placed in the plating bath and connected to an electrochemical workstation (Chi 600D). Negative potential was applied on the RDE, where the electrodeposition of Cu took place. As shown in Fig. 1, the RDE was connected to the negative pole of the power supply, while both the RE and the CE was connected to the positive pole. The CA tests were set at different potentials with a fixed rotation speed of 600 rpm.

At the beginning, the CA test was carried out with the base electrolyte, under condition of a set working potential. After a response time (normally 40 s), the current response of the electrodeposition system gradually became stable. Once a steady-state current was attained, the additives were rapidly injected into the bulk using a syringe. As additives with inhibition effect on electrodeposition rate injecting into the electrolyte, the current response would drop with a certain degree. When a new steady state of the current response was attained, it was recorded. The drop in current response indicated the inhibition effect of the certain amount of the additive. The injection operation was repeated until the saturated value of the current response against the concentration of the additives was obtained. Two types of experiments were conducted: (a) single additive injection, (b) multiple additives injection.

### Individual additive

As shown in Table 1, the CA tests for the suppressor were performed potentiostatically at −0.6 V, −0.55 V, −0.53 V, and −0.5 V, and the concentration of suppressor was varied from 0 ml/L to 10 ml/L. Considering the possibility that the suppressor had better inhibiting effect at high potential, additional investigations were made at −0.53 V and −0.5 V with the concentration ranged from 0 ml/L to 1 ml/L. Similar to the suppressor, CA tests were carried out to study the effects of the leveler and the accelerator on the copper electroplating. Table 2 and Table 3 list the experimental parameters of 4 groups of tests for leveler and 3 groups for accelerator, respectively. In the tables, E (V) denotes over-potential; ${C}_{sup}$ denotes the concentration of the suppressor; ${C}_{acc}$ denotes the concentration of the accelerator and ${{\rm{C}}}_{lev}$ denotes the concentration of the leveler.

**Table 1:** CA test parameters for the suppressor.

E (V)	${{\boldsymbol{C}}}_{{\boldsymbol{\sup }}}$ (ml/L)
−0.6	0, 1, 2, 3, 4, 5, 6, 7, 8, 9, 10
−0.55	0, 1, 2, 3, 4, 5, 6, 7, 8, 9, 10
−0.53	0, 0.4, 0.75, 1, 2, 3, 4, 5, 6, 7, 8, 9, 10
−0.5	0, 0.2, 0.4, 0.6, 1, 2, 3, 4, 5, 6, 7, 8, 9, 10

**Table 2:** CA test parameters for the leveler.

E (V)	${{\boldsymbol{C}}}_{{\boldsymbol{lev}}}$ (ml/L)
−0.6	0, 4, 8, 12, 16, 20, 24, 28, 32
−0.55	0, 4, 8, 12, 16, 20, 24, 28, 32
−0.53	0, 4, 8, 12, 16, 20, 24, 28, 32
−0.5	0, 4, 8, 12, 16, 20, 24, 28, 32

**Table 3:** CA test parameters for the accelerator.

E (V)	${{\boldsymbol{C}}}_{{\boldsymbol{acc}}}$ (ml/L)
−0.6	0, 2, 4,5, 6, 8, 10, 12
−0.55	0, 2, 4, 6, 8, 10, 12
−0.5	0, 2, 4, 5, 6, 8, 10, 12

### Interaction of additives

In order to investigate the interaction of additives in the copper electrodeposition, CA tests with multiple additives were performed. Before the CA test commence, one kind of additive with a certain concentration was premixed into the electrolyte; another kind of additive was later injected into the electrolyte during the CA test process. As shown in Table 4, for the study of suppressor and accelerator interaction, the suppressor with concentration of 6.2 ml/L was premixed into the electrolyte, and when the CA test started, the concentration of the accelerator into the electrolyte was increased from 0 ml/L to 12 ml/L by injection. The interaction experiments were conducted each other in pairs in a similar fashion, as shown in Tables 5 and 6.

**Table 4:** CA test parameters for the interaction between the suppressor and the accelerator.

E (V)	${{\bf{C}}}_{{\boldsymbol{\sup }}}$ (ml/L) (premixed)	${{\bf{C}}}_{{\bf{a}}{\bf{c}}{\bf{c}}}$ (ml/L) (inject)
−0.6	6.2	0, 2, 4, 6, 8, 10, 12
−0.55	6.2	0, 2, 4, 6, 8, 10, 12
−0.53	6.2	0, 2, 4, 6, 8, 10, 12
−0.5	6.2	0, 2, 4, 6, 8, 10, 12

**Table 5:** CA test parameters for the interaction between the accelerator and the leveler.

E (V)	${{\bf{C}}}_{{\bf{a}}{\bf{c}}{\bf{c}}}$ (ml/L) (premixed)	${{\bf{C}}}_{{\boldsymbol{lev}}}$ (ml/L) (inject)
−0.6	1	0, 4, 8, 12, 16, 20, 24, 28, 32
−0.6	5	0, 4, 8, 12, 16, 20, 24, 28, 32
−0.5	1	0, 4, 8, 12, 16, 20, 24, 28, 32
−0.5	5	0, 4, 8, 12, 16, 20, 24, 28, 32

**Table 6:** CA test parameters for the interaction between the suppressor and the leveler.

E (V)	${{\bf{C}}}_{{\boldsymbol{\sup }}}$ (ml/L) (premixed)	${{\bf{C}}}_{{\boldsymbol{lev}}}$ (ml/L) (inject)
−0.6	6.2	0, 4, 8, 12, 16, 20, 24, 28, 32
−0.5	0.2	0, 4, 8, 12, 16, 20, 24, 28, 32

### Micro via filling by electrodeposition

Based on the results of the electrochemical analysis, experiments of micro via filling by electrodeposition were conducted. The effects of singular additive and multiple additives on micro via filling were studied in separate experiments (Table 7). The base electrolyte was used in the same way as the electrochemical analysis experiments, the current density was set with 0.5 A/dm^2^, and the temperature was maintained at 25 °C.

**Table 7:** Experimental parameters of electrodeposition for micro via filling.

Experiments	Accelerator (ml/L)	Leveler (ml/L)	Suppressor (ml/L)
A	0	0	0
B	5	0	0
C	0	8	0
D	0	0	5
E	5	8	0
F	5	0	5
G	0	8	5
H	5	8	5
I	5	12	10

## Results and discussion

Utilizing the parameters provided in Tables 1–6, the experiments were repeated three times. The average of the results was calculated, and error bar was derived. The effects of individual additive and additives’ interaction on the exchange current density of the electrochemical system are discussed. Experiments of micro via filling by electrodeposition were conducted, and non-defect filling of the micro via was obtained.

### Effect of individual additive

#### Effect of suppressor

**Figure 2:**
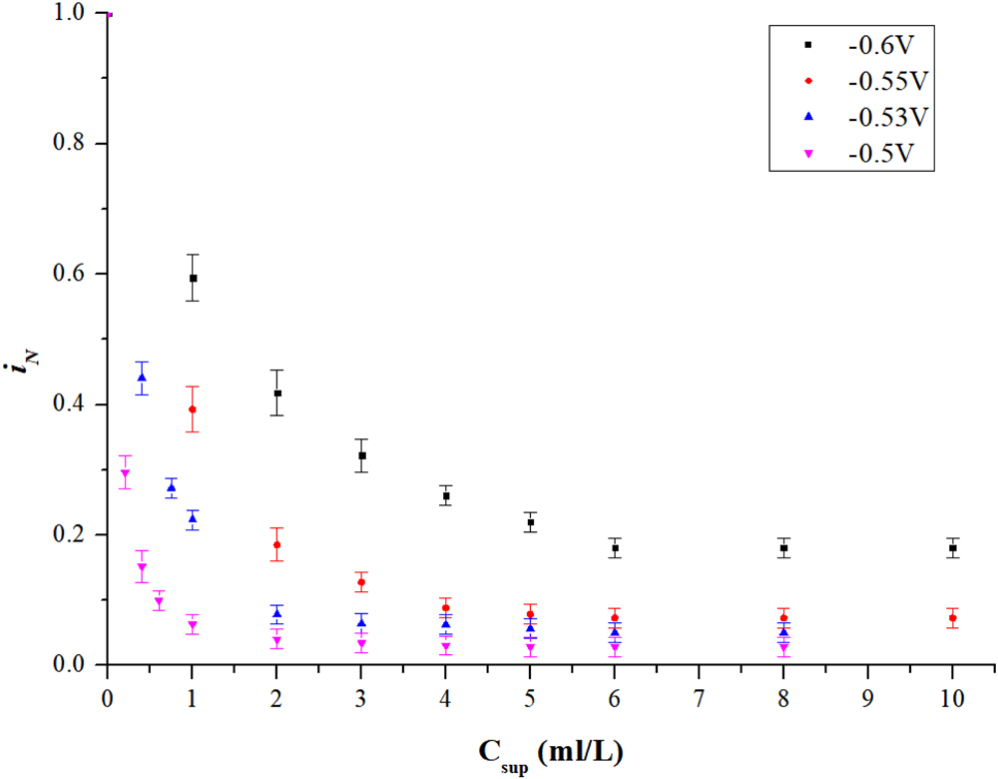
Effect of singular suppressor.

Figure 2 presents the CA measurement results of singular suppressor under different potential (−0.6 V, −0.55 V, −0.53 V, and −0.5 V). The normalized current density (${i}_{N}$) indicating the degree of inhibition of additives on the current, is defined as:

1 $${i}_{N}=\frac{{i}_{add}}{{i}_{0}}$$

where ${i}_{0}$ and ${i}_{add}$ represent the current density in base electrolyte and electrolyte added additives (singular suppressor in this section), respectively. According to the experimental results in Fig. 2, conclusions can be drawn as follows: (1) higher suppressor concentration leads to lower ${i}_{N}$. As the suppressor concentration increased, inhibition ability of the suppressor is obviously enhanced until a saturated state is attained. (2) the inhibition behaviour of the suppressor has a significant dependence on the over-potential, which results in better performance of inhibition at higher potential. The ${i}_{N}$ under a lower level potential of −0.6 V declines slowly at the beginning and reaches a saturation at the suppressor concentration around 6 ml/L. As the over-potential becomes higher (less negative), the ${i}_{N}$ drops faster (more sensitive to the concentration of the additive) and becomes stable at an earlier stage (saturated with low concentration the additive). Especially, the ${i}_{N}$ under the potential of −0.5 V is dramatically inhibited by 94% as the suppressor concentration increases from 0 ml/L to 0.4 ml/L, and reaches an early steady state at suppressor concentration of around 3 ml/L. 3) in the stable state, the maximum inhibition of the suppressor is related to potential. Compared with the test without any additives (${{\rm{C}}}_{\sup }=0$), ${i}_{N}$ in suppressor injection test is inhibited by 82% under the potential of −0.6 V (${{\rm{C}}}_{\sup }=8$ ml/L), and 97% under the potential of −0.5 V (${{\rm{C}}}_{\sup }=8$ ml/L).

#### Effect of leveller

**Figure 3:**
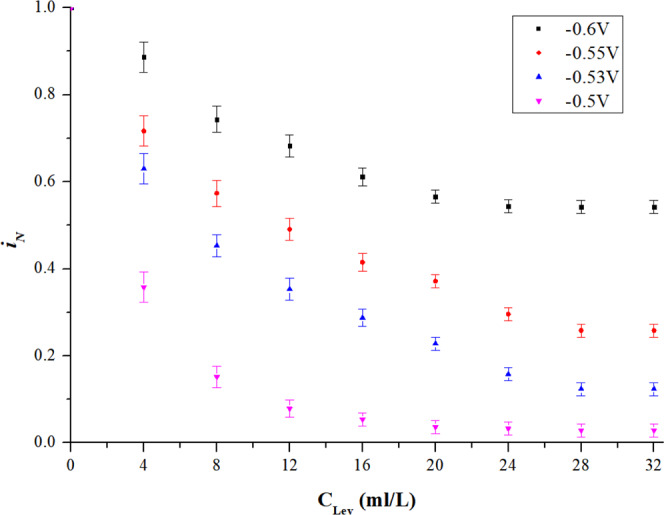
Effect of singular leveler.

As shown in Fig. 3, conclusions can be drawn as follows: 1) the leveler can also inhibit ${i}_{N}$, but the effect is apparently weaker than the suppressor. The downtrend of ${i}_{N}$ is slow and the curves are flattened after 20 ml/L. 2) similar to the suppressor, the effect of leveler also shows potential dependence. Inhibition performance is much better at higher potential (less negative), where the decrement of ${i}_{N}$ is more sensitive to the concentration of the leveler and becomes stable with low concentration of the leveler. 3) in stable state, inhibition related to potential could not be fully attained. The ${i}_{N}$ is inhibited by 46% at −0.6 V, and 97% at −0.5 V.

#### Effect of accelerator

**Figure 4:**
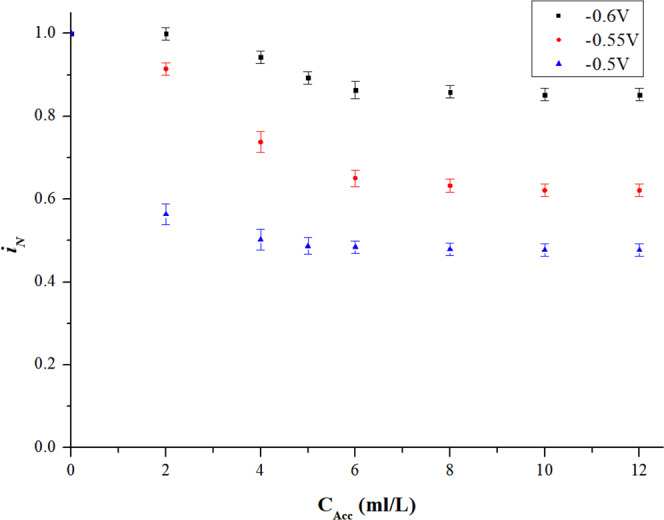
Effect of singular accelerator.

The experiment results are shown in Fig. 4. It can be concluded that: (1) with increasing concentration, the accelerator has an insignificant inhibition effect, which is much weaker than suppressor or leveler. Especially, the ${i}_{N}$ at −0.6 V has little change when the concentration is increased to 2 ml/L. The value of ${i}_{N}$ declines as the accelerator becomes more concentrated, until a saturated state is attained. (2) the downtrend of ${i}_{N}$ also shows potential dependence, similar to the suppressor and the leveler. Higher potential (less negative) contributes to stronger inhibition effect which is more sensitive to the concentration of the additives than under condition of lower potential (more negative). (3) the maximum inhibition effect of accelerator is also related to potential. In the stable state, the ${i}_{N}$ is inhibited by 15% at −0.6 V, and 52% at −0.5 V.

### Effect of additives interaction

#### Interaction of suppressor and accelerator

In the interaction tests of the suppressor and the accelerator, the concentration of the suppressor was fixed at 6.2 ml/L, while the concentration of the accelerator was increased from 0 ml/L to 12 ml/L. From the previous analysis, it is known that the accelerator shows much weaker inhibition ability than the suppressor. By contrast, the accelerator has a relative promoting effect on electroplating comparing to the suppressor.

**Figure 5:**
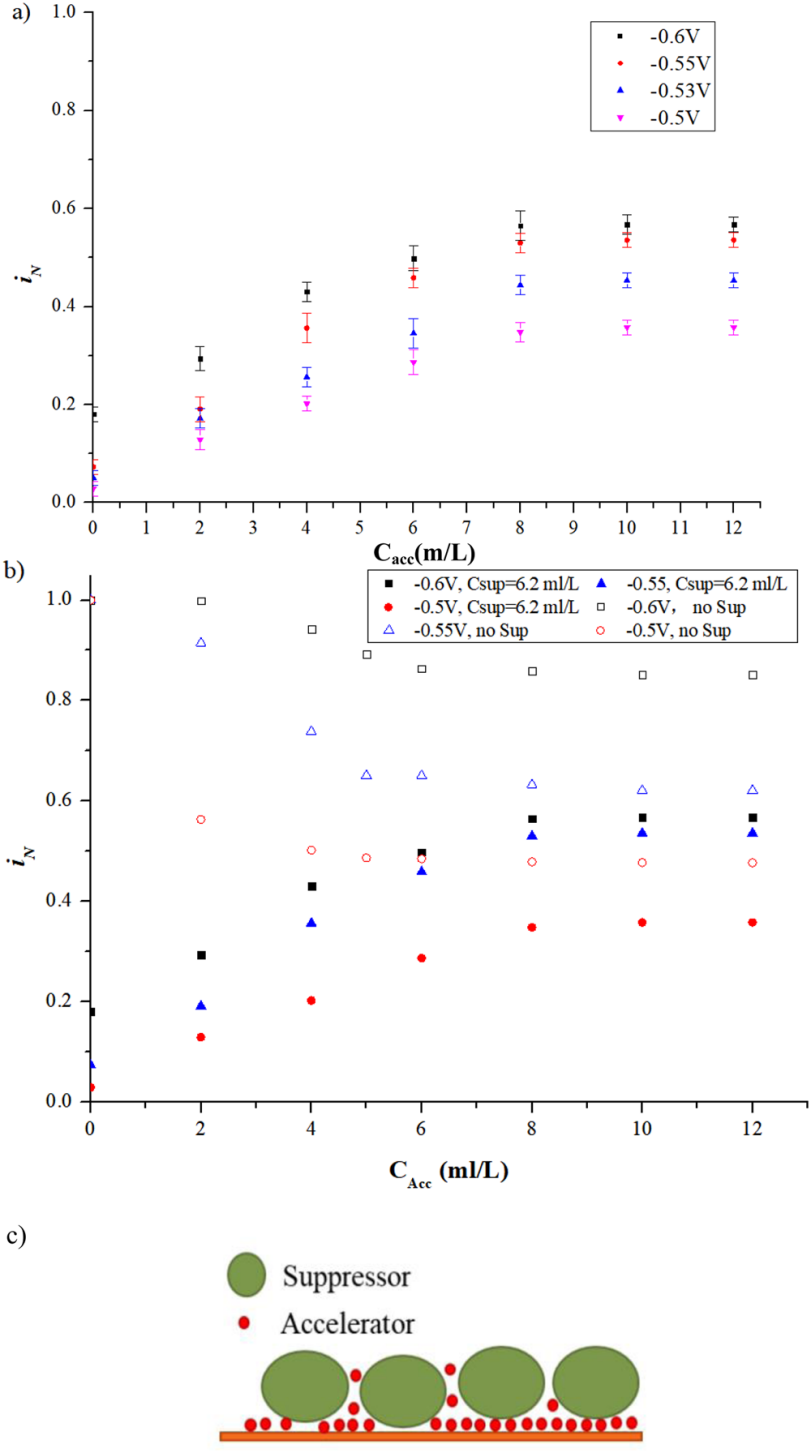
Interaction of accelerator and suppressor (**a**) CA measurement results of accelerator added into electrolyte premixed suppressor of 6.2 ml/L; (**b**) Comparison of the results of the singular accelerator and the accelerator with premixed suppress of 6.2 ml/L; (**c**) Schematic of the interaction between suppressor and accelerator.

Figure 5 shows the experimental results of the interaction of accelerator and suppressor. Based on the CA measurement results of the suppressor, when only the accelerator added into the electrolyte, it showed a slight inhibition effect on the exchange current (Fig. 4) and when the accelerator added into the electrolyte premixed with suppresser (Fig. 5a,b)), the accelerator showed a relative accelerating effect on ${i}_{N}$. The reasons are that (1). The suppressor showed much stronger inhibition effect on ${i}_{N}$ than the accelerator; (2). As shown in Fig. 5c), the accelerator displaces the suppressor which adsorbed on the cathode surface in advance resulting an increasing of ${i}_{N}$. As a result, the accelerator showed a relative accelerating effect when it added into the electrolyte premixed with suppresser.

As shown in Fig. 5a), the value of ${i}_{N}$ climbs with the increasing concentration of the accelerator added into the electrolyte that is premixed a fixed concentration of the suppressor (6.2 ml/L). In order to clearly describe the interaction behaviour between the suppressor and the accelerator, Fig. 5b presents a comparison of ${i}_{N}$ between condition of the singular accelerator and condition affected by the interaction of accelerator and suppressor. It can be found from Fig. 5b that the value of ${i}_{N}$ of the interaction system increases along with the accelerator gradually adding into the electrolyte premixed with suppressor. The explanation is that the suppressor adsorbed on the cathode surface is displaced by the accelerator when the accelerator is injected into the electrolyte premixed with the suppressor. As the concentration of the accelerator increased, displacement of the suppressor by the accelerator is keeping going, leading to less suppressor molecules and more accelerator molecules adsorbed on the cathode surface. Hence, the value of ${i}_{N}$ of the interaction system is increased. In addition, keeping increasing the concentration of the accelerator in the electrolyte, the value of ${i}_{N}$ of the interaction system gradually approaches saturation. However, the saturation of the interaction system was still lower than that of the individual accelerator. This experimental result reveals that the suppressor adsorbed on the cathode surface cannot be completely replaced by the accelerator. As shown in Fig. 5b, the saturated value of ${i}_{N}$ of the interaction system is lower than that of the singular accelerator system, although they are very close indicating that there were still some residual suppressors left on the cathode surface. Under potential of −0.6 V, 0.55 and 0.5 V, the saturated value of ${i}_{N}$ of the interaction system are 0.57, 0.54 and 0.36, respectively, while that of the singular accelerator system are 0.85, 0.62 and 0.48, respectively. As schematically shown in Fig. 5c, due to the much smaller size of the accelerator than that of the suppressor, the accelerator will penetrate the gaps between the suppressor and eventually displace the suppressor on the cathode surface. The possible explanation is that the accelerator has an easier and stronger bond with the Cl^−^ adsorbed on the cathode surface than the suppressor. In the interaction system, the accelerator broke the bond between suppressor and Cl^−^, leading to suppressor’s desorption and displacement by accelerator.

#### Interaction of accelerator and leveller

**Figure 6:**
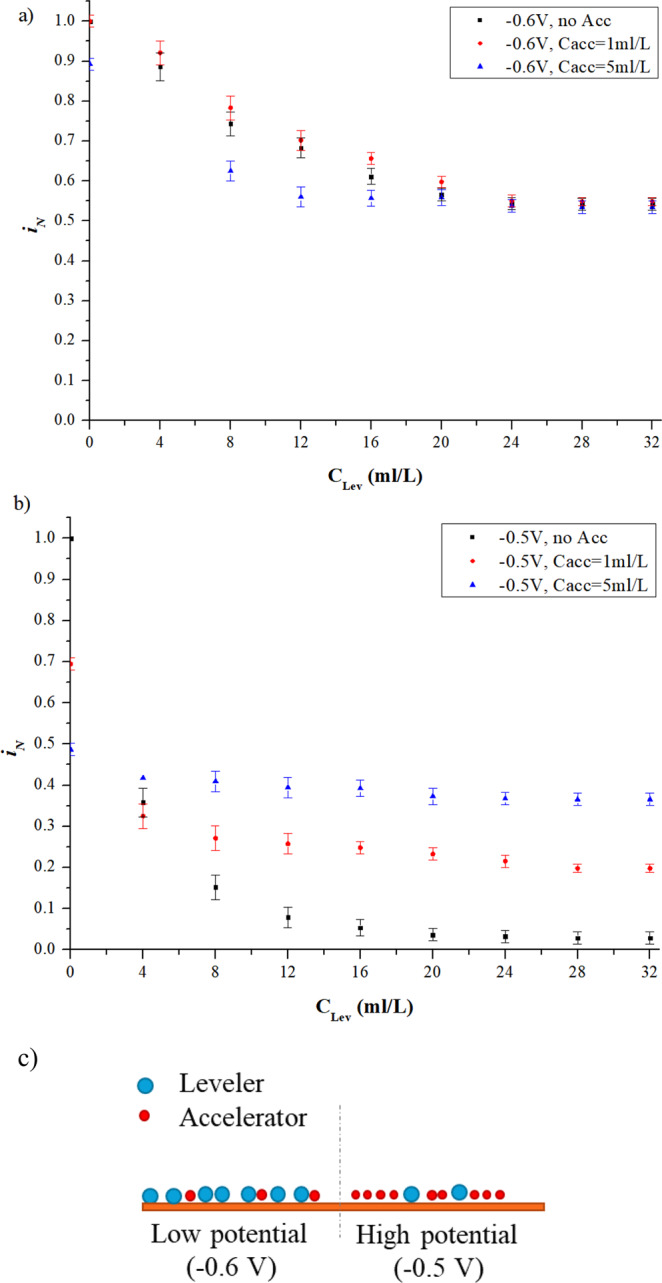
Interaction of accelerator and leveler. a) CA measurement results of leveler injected into electrolyte premixed accelerator under low potential (−0.6 V); b) CA measurement results of leveler injected into electrolyte premixed accelerator under high potential (−0.5 V); c) Schematic of the interaction between leveler and accelerator.

As shown in Fig. 6a, with the over-potential fixed at −0.6 V, the downtrend and stable value of ${i}_{N}$ in the interaction systems with fixed accelerator concentration of 1 ml/L and 5 ml/L both shows good agreement with the singular leveler system, indicating dominance of the effect of leveler in the inhibition behaviour. However, when the potential is set at −0.5 V, as shown in Fig. 6b, the value of ${i}_{N}$ at stable state in the interaction systems is much higher than that of singular leveler system, indicating that the accelerator plays a major role in the interaction systems. Therefore, it can be concluded that the accelerator and the leveler have a competitive adsorption relationship in the electroplating process: (1) the leveler overcomes the accelerator at low potential (−0.6 V); (2) the accelerator overcomes the leveler at high potential (−0.5 V). The competitive adsorption relationship between the leveler and the accelerator is schematically shown in Fig. 6c. Under low potential (−0.6 V), the cathode surface is dominated by the leveler; while under high potential (−0.5 V), the cathode surface is dominated by the accelerators.

#### Interaction of suppressor and leveller

**Figure 7:**
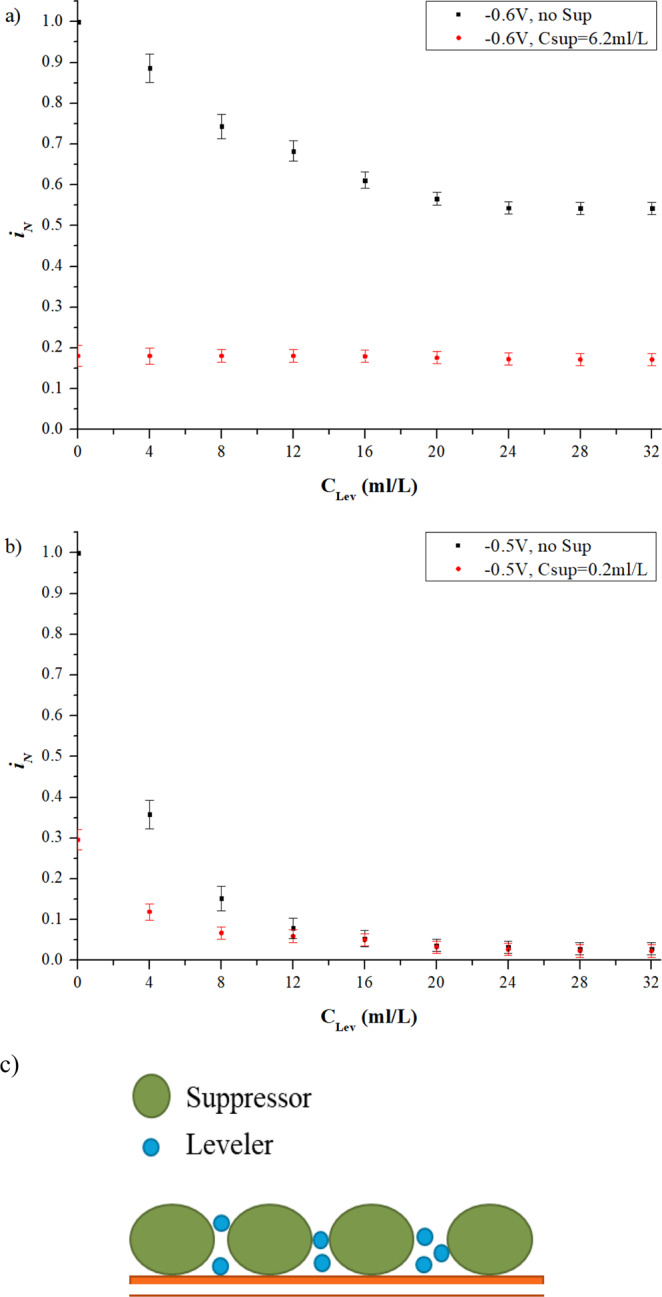
Interaction of leveler and suppressor. (**a**) CA measurement results of leveler injected into electrolyte premixed suppressor under low potential; (**b**) CA measurement results of leveler injected into electrolyte premixed suppressor under high potential; (**c**) Schematic of the interaction between suppressor and leveler.

Two groups of experiments were conducted to study the interaction of suppressor and leveler, as given in Table 6. The first group was investigated with relative low over-potential and high concentration of the suppressor, which were fixed at −0.6 V and 6.2 ml/L respectively, with the increase of leveler from 0 ml/L to 32 ml/L. Figure 7a shows the comparison of ${i}_{N}$ between singular leveler and interaction system of leveler and suppressor under low potential, from which it can be seen that the increasing leveler has nearly no influence on the ${i}_{N}$, which is defined in Eq. (1) where ${i}_{add}$ represented the current density in suppressor and leveler interaction system. Another group was conducted with relative high over-potential and low concentration of the suppressor, fixed at −0.5 V and 0.2 ml/L, respectively. As shown in Fig. 7b, ${i}_{N}$ in the interaction system is evidently inhibited by the increasing concentration of leveler and becomes stable at 0.023 (97.7% is inhibited), much less than that in the singular suppressor system at 0.29 (71% is inhibited). It can be concluded that under high potential (−0.5 V) and low concentration of suppressor (0.2 ml/L), interaction of suppressor and leveler has a synergistic effect for electroplating inhibition, as schematically shown in Fig. 7c. Unlike the accelerators, the levelers are not tend to displace the suppressor from the cathode, but fill the gaps among the suppressors. The coverage rate of the cathode is improved by the synergistic effect of the leveler and the suppressor, leading to less *i*_*N*_.

### Micro via filling by electrodeposition

**Figure 8:**
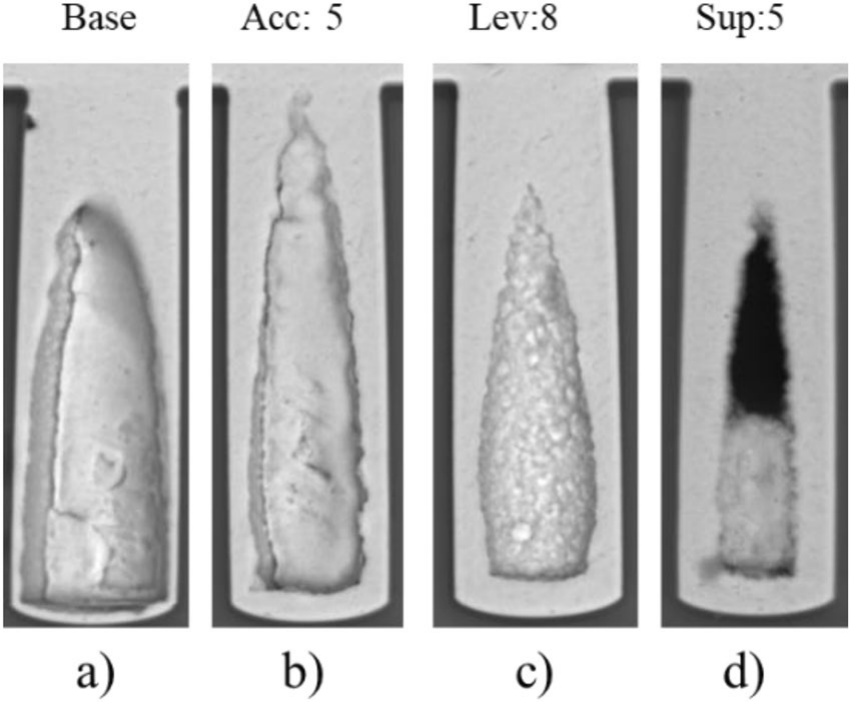
Micro via filled by electrodeposition with singular additives.

Based on the results of the electrochemical analysis presented above, experiments of micro via filling by electrodeposition were conducted (Table 7). As shown in Fig. 8, the cross section of the samples was observed by SEM. Figure 8a shows the micro via filled without assistance of additives, where a large void is formed in the micro via, and very limited Cu has been deposited at the bottom of the micro via. However, if additives were added into the electrolyte, the defects can be alleviated. As shown in Fig. 8b–d, the size of the voids in the micro vias after the filling process using different type of additive is decreased in turn.

The filling ratio is obtained, which is defined as the percentage of the filled area against the whole area of the micro via. With no additive adding into the electrolyte (base), the filling ratio is 46.8% (Fig. 8a)). With accelerator (5 ml/L), leveler (8 ml/L) and suppressor (5 ml/L) adding into the electrolyte, the filling ratio are 52.4% (Fig. 8b)), 61.1% (Fig. 8c)) and 73.6% (Fig. 8d)), respectively. The results indicate that stronger inhibition effect of the additives leads to higher filling ratio (less void).

The results show good agreement with the results of the electrochemical analysis for the additives, i.e. the accelerator shows slight inhibition effect on electrodeposition, while the leveler is medium and the suppressor is strong.

**Figure 9:**
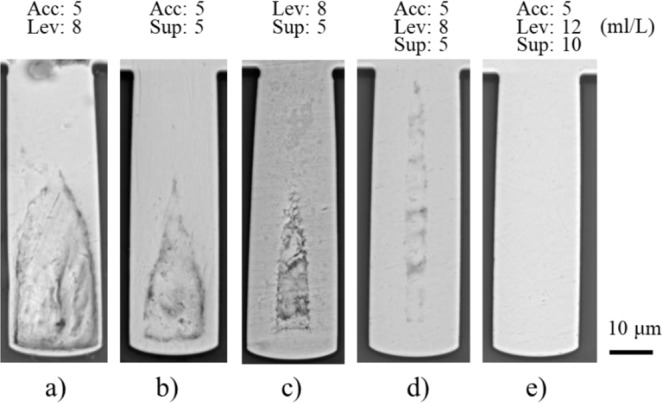
Micro via filled by electrodeposition with multiple additives.

The interaction effects of additives on micro via filling through electrodeposition are presented in Fig. 9a–c). When accelerator added into the electrodeposition process, the micro vias filled with Acc and Lev (Fig. 9a)) and with Acc and Sup (Fig. 9b)) show larger voids at the deep bottom. Their filling ratios are 57.0% and 76.3%, respectively. While under the conditions with Lev and Sup (no Acc), the void in the micro via becomes smaller (Fig. 8c)), with filling ratio of 82.8%. A possible explanation is that the inhibition effects of the suppressor and the leveler were undermined by the accelerator, leading to the sealing of the voids in a shorter time and less Cu deposition at the bottom of the micro via. The micro via shown in Fig. 9c) was filled with addition of both suppressor and leveler in the bath, which shows a smaller void at the bottom of the micro via than that of Fig. 8c,d. This is probably caused by the synergic inhibition effect of the suppressor and the leveler in the electrodeposition process.

Figure 9d presents the result of micro via filled by adding the three additives in the bath. It can be seen from the result that the defect in the micro via is greatly alleviated (filling ratio of 87.2%), compared with the results shown in Figs. 8a–d and 9a–c). However, a small seam still exists in the micro via (Fig. 9d)). In order to solve this problem, a feasible solution is to further inhibit the Cu depositing rate near the opening region of the micro via. As shown in Fig. 9e), the micro via has been filled without defect (filling ratio of 100%), where the accelerator (5 ml/L), the leveler (12 ml/L) and the suppressor (10 ml/L) were added in the bath.

## Conclusions

In this paper, effects of singular additive and additives’ interaction on electroplating were investigated by experimental analysis. The conclusions are summarized as follows:

1. In the singular additive system, the suppressor, leveler and accelerator all show different degrees of inhibition ability, and the inhibition behaviour is potential dependent;
2. Interaction of the suppressor and the accelerator indicates that the suppressor absorbed on the cathode surface is gradually displaced by the accelerator as the concentration of the accelerator increases;
3. The accelerator and the leveler have a competitive relationship with respect to adsorption amount on the cathode surface, which indicates that the leveler tends to replace the accelerator at low potential while the accelerator tends to replace the leveler at high potential;
4. The suppressor and the leveler have a synergistic effect for electroplating inhibition, especially under high potential and low suppressor concentration.
5. Micro vias have been filled by Cu electrodeposition, with addition of singular additive and multiple additives in the bath, and non-defect filling of the micro via has been achieved.
